# Cannabinoids Reduce Markers of Inflammation and Fibrosis in Pancreatic Stellate Cells

**DOI:** 10.1371/journal.pone.0001701

**Published:** 2008-02-27

**Authors:** Christoph W. Michalski, Milena Maier, Mert Erkan, Danguole Sauliunaite, Frank Bergmann, Pal Pacher, Sandor Batkai, Nathalia A. Giese, Thomas Giese, Helmut Friess, Jörg Kleeff

**Affiliations:** 1 Department of Surgery, Technische Universität München, Munich, Germany; 2 Department of General Surgery, University of Heidelberg, Heidelberg, Germany; 3 Institute of Pathology, University of Heidelberg, Heidelberg, Germany; 4 Section of Oxidative Stress Tissue Injury, Laboratory of Physiologic Studies, National Institutes of Health, National Institute on Alcohol Abuse and Alcoholism (NIAAA), Bethesda, Maryland, United States of America; 5 Institute of Immunology, University of Heidelberg, Heidelberg, Germany; Copenhagen University Hospital, Denmark

## Abstract

**Background:**

While cannabinoids have been shown to ameliorate liver fibrosis, their effects in chronic pancreatitis and on pancreatic stellate cells (PSC) are unknown.

**Methodology/Principal Findings:**

The activity of the endocannabinoid system was evaluated in human chronic pancreatitis (CP) tissues. In vitro, effects of blockade and activation of cannabinoid receptors on pancreatic stellate cells were characterized. In CP, cannabinoid receptors were detected predominantly in areas with inflammatory changes, stellate cells and nerves. Levels of endocannabinoids were decreased compared with normal pancreas. Cannabinoid-receptor-1 antagonism effectuated a small PSC phenotype and a trend toward increased invasiveness. Activation of cannabinoid receptors, however, induced de-activation of PSC and dose-dependently inhibited growth and decreased IL-6 and MCP-1 secretion as well as fibronectin, collagen1 and alphaSMA levels. De-activation of PSC was partially reversible using a combination of cannabinoid-receptor-1 and -2 antagonists. Concomitantly, cannabinoid receptor activation specifically decreased invasiveness of PSC, MMP-2 secretion and led to changes in PSC phenotype accompanied by a reduction of intracellular stress fibres.

**Conclusions/Significance:**

Augmentation of the endocannabinoid system via exogenously administered cannabinoid receptor agonists specifically induces a functionally and metabolically quiescent pancreatic stellate cell phenotype and may thus constitute an option to treat inflammation and fibrosis in chronic pancreatitis.

## Introduction

The management of chronic pancreatitis still remains a clinical challenge, with no definite medical cure and only symptomatic treatment available for this disease[1]–[3]. In some cases, surgical resection of the inflammatory mass (usually localized in the pancreatic head) may permanently relieve symptoms[4]. Histologically, areas of fibrosis (deposits of extracellular matrix (ECM) proteins) are found which may contain clusters of mononuclear cell infiltration[5]–[7]. Enlarged nerves may be invaded by mononuclear cells, potentially leading to neural damage, which may in part explain the severe pain syndrome[8], [9]. As a consequence, endocrine and exocrine functions of the pancreas are progressively lost, ultimately resulting in a scarred pancreas without its physiological functions.

In the past years, pancreatic stellate cells (activated myofibroblasts; PSC) have been identified as major determinants of pancreatic fibrosis: they have been shown to be the major source of extracellular matrix production[10], [11] and to stringently control the balance of ECM secretion and digestion by producing matrix metalloproteinases and their corresponding inhibitors[12]. PSC also modulate the local immune reaction by production and secretion of cytokines and chemokines as well as by their phagocytic activity[13]–[17]. However, the pathobiology of pancreatic fibrogenesis/inflammation and the interplay between stellate cells[18]–[20], immune cells and nerves is poorly understood, and currently no potentially curative medical treatment is available.

Comparable with liver cirrhosis, prevention of loss of functional pancreatic parenchyma by controlling and resolving the overt scarring reaction to an inflammatory stimulus may constitute a therapeutic approach. Although a number of substances have been identified so far which were initially promising in ameliorating or even reversing the disease, none of these was clinically proven to exert such beneficial properties[21]–[23].

Besides the well-known central-nervous analgesic properties of exogenously administered cannabinoids, the endocannabinoid system (ECS) and its changes in pathological states have recently attracted considerable attention[24], [25]. Particularly, cannabinoids' immune-modulatory function and their influence on lymphocytes constitute a basis for their use in a wide variety of inflammatory diseases[26]–[30]. Besides these well-studied effects, (endo-)cannabinoids have recently been shown to influence liver fibrogenesis through various mechanisms. Siegmund and co-workers[31], [32] have shown that the endocannabinoid anandamide induces necrosis in hepatic stellate cells independent of CB1 and CB2 receptors. In contrast to these results, Julien et al.[33] have found in experimentally induced liver cirrhosis that an activation of the CB2 receptor on hepatic stellate cells leads to apoptosis and attenuated liver fibrosis progression. Teixeira-Clerk and co-authors[34] have proposed CB1 antagonism as a new strategy to treat liver fibrosis. Altogether, these results point towards a potential use of cannabinoids as substances to ameliorate or even revert liver fibrogenesis. However, the exact mechanism of how (and particularly in which disease stage) either CB receptor activation or antagonism may be useful in attenuating chronic liver cirrhosis remains to be determined.

In chronic pancreatitis, activity of the endocannabinoid system and effects of exogenously administered cannabinoids have not been analyzed so far. In this study, we evaluated levels of endocannabinoids and their receptors as well as the potential function of cannabinoid activation and antagonism by synthetic cannabinoid derivatives and their respective antagonists in human chronic pancreatitis tissues and CP-derived PSC.

## Materials and Methods

### Patients and tissue collection

Pancreas tissues were obtained intraoperatively from patients undergoing resection for chronic pancreatitis (21 men, 19 women). Normal pancreas tissue samples were collected within the organ donor program at Heidelberg University hospital whenever there was no suitable recipient for organ transplantation (n = 20). All patients were informed, and written consent was obtained. The studies were approved by the Ethics Committee of the University of Heidelberg (Germany).

### Immunohistochemistry of human pancreatic tissues

CB1- and CB2-receptors were localized in the human pancreas using immunohistochemistry. Rabbit anti human-CB1 and anti human-CB2 antibodies (Cayman Chemical, Ann Arbor, MI, USA) were used at a dilution of 1∶150 and 1∶300, respectively. Specificity was checked by performing pre-adsorption of the primary antibody with the corresponding blocking peptide for 1 hour at 37°C.

### Endocannabinoid level measurements

Levels of anandamide (AEA), 1-arachidonoylglycerol (1-AG) and 2-arachidonoylglycerol (2-AG) in frozen human pancreas samples (normal pancreas: n = 6; chronic pancreatitis: n = 6) were determined by liquid chromatography/mass spectrometry as described previously [35].

### Reagents

Ham's F12 medium, DMEM, trypsin-EDTA and penicillin-streptomycin were purchased from Invitrogen (Karlsruhe, Germany); amphotericin B 250 µg/ml was purchased from PAA Laboratories (Pasching, Germany); fetal calf serum (FCS) was purchased from PAN Biotech (Aidenbach, Germany); the enhanced chemoluminescence (ECL) immunoblotting detection reagents were obtained from Amersham Biosciences (Buckinghamshire, UK); Mini EDTA-Free Protease inhibitor was purchased from Roche Molecular Biochemicals (Basel, Switzerland); and the BCA protein assay was from Pierce Chemical Co. (Rockford, IL, USA) and the MTT test reagent was purchased from Sigma Aldrich (Taufkirchen, Germany).

### Isolation of human pancreatic stellate cells

Human PSC isolation and culture were performed as described by Bachem et al.[11] using the outgrowth method. For our experiments, cell populations between passage 3 and 5 were used. A 1∶1 (vol/vol) mixture of low glucose (1000 mg/L) DMEM with Ham's F12 medium supplemented with 20% FCS, L-glutamine (2 mM), penicillin/streptomycin, and amphotericin B was the standard growth medium, whereas for pharmacology experiments, 1% FCS was used.

### Immunocytochemistry

Immunocytochemistry was performed as described previously[36]. Cells were seeded in 20% FCS. Anti-CB1-receptor and anti-CB2-receptor antibodies were diluted 1∶300 in Antibody Diluent (DakoCytomation, Hamburg, Germany). Specificity controls included pre-adsorption of the primary antibody with the corresponding blocking peptide (1∶1) for 1 hour at 37°C.

### Cannabinoid treatment and proliferation assays

PSC were plated on the bottom of 24-well plates at densities of 40,000/well in 500 µl DMEM/Ham's F12 (1/1, v/v) in the presence of 20% FCS for 24 hours. Subsequently, the medium was changed to DMEM/Ham's F12 with 1% FCS. After overnight incubation, drugs were added at concentrations of 1.25, 2.5 and 5 µM. WIN55,212-2 (WIN; Tocris Cookson Ltd., Avonmouth, UK) was dissolved in DMSO and further diluted in ethanol (EtOH). Specific CB1-receptor and CB2-receptor antagonists (AM251 and AM630, respectively) were dissolved in EtOH. Controls included DMSO/EtOH at a dilution of 1∶1000. After 48 hours of incubation, proliferation was determined using MTT tests, as described previously[37]. These experiments were repeated five times, whereas experiments for determination of WIN-specificity (pre-incubation with AM251 and/or AM630 before addition of WIN) were repeated twice. To exclude that potential anti-proliferative effects of cannabinoids influenced evaluation of cytokine and ECM protein secretion, treatment was begun when the cells reached 100% confluency.

### Apoptosis assays

Cells were grown to 70–80% confluency in medium containing 20% FCS before the FCS concentration was reduced to 1%. After 24 hours of incubation, drugs were added for another 24 hours, as described for the proliferation assays. Single cell suspensions were obtained using trypsin-EDTA. Subsequently, the Guava Nexin™ kit (Guava Technologies, Hayward, CA, USA) was used to determine the number of early and late apoptotic cells according to the manufacturer's instructions.

### LDH analysis

Lactate dehydrogenase (LDH) in cell culture supernatants was measured in the central clinical laboratories at the University of Heidelberg using an ADVIA2400 machine (Siemens, Erlangen, Germany).

### Invasion assay

Matrigel™-coated invasion chambers (BioCoat Matrigel Invasion Chamber, BD Biosciences, Heidelberg, Germany) were rehydrated for 2 hours in serum-free medium at 37°C and 5% CO_2_. PSC were trypsinized and resuspended in serum-free medium at a concentration of 2.5×10^4^ cells per insert. 675 µl serum-free medium was added to the inner wells. Cannabinoid receptor antagonists AM251 and AM630, a combination of both, or the respective controls were added to the wells and the inserts to a final concentration of 2.5 µM. Following 30 min of incubation, the synthetic cannabinoid agonist WIN55,212-2 was also added to all wells and inserts (final concentration of 2.5 µM). At the end of the 24-hour incubation period, supernatants were aspirated and the upper part of the membrane was cleaned with cotton swabs. Cells were then fixed with 75% methanol and 25% acetone for 30 minutes and subsequently stained with toluidine blue. The inserts were rinsed in distilled water to remove excess stain. Finally, the membranes were placed on glass slides and invaded cells were counted.

### Immunofluorescence assays

Immunofluorescence assays were performed as previously described [38]. Briefly, 1×10^4^ PSC per well were seeded on teflon™-coated slides (Erie Scientific Company, Portsmouth, NH, USA) in 20% FCS-containing medium, and were incubated for 24 hours before the medium was changed to 1% FCS. Upon further incubation for 24 hours, the cannabinoid agonist WIN55,212-2, the CB1-receptor antagonist AM251, or the CB2-receptor antagonist AM630 was added at a final concentration of 5 µM. After 48 hours, cells were fixed with 4% paraformaldehyde, permeabilized with 1% Triton-X, and incubated with phalloidin (1∶500) for 20 minutes. To counterstain the nuclei, DAPI stain was added at a 1∶1000 dilution.

### ELISA

To determine levels of MCP-1, IL-6 and TGF-beta in cell culture supernatants, commercial solid phase sandwich ELISA kits (BD OptEIA™, BD Biosciences, Heidelberg, Germany) were used according to the manufacturer's instructions. MMP-2 levels were determined using an ELISA kit obtained from R&D Systems (Minneapolis, MN, USA) according to the instructions of the manufacturer.

### Immunoblot analysis (ECM proteins)

For immunoblot analyses, cell culture supernatants or cell lysates (cells treated with 2.5 µM of WIN, AM251 and/or AM630; or WIN (2.5 µM)+/−AM251/AM630 (5 µM) as indicated in “proliferation assays”) were processed as described [38]. Cells were grown to reach 100% confluency before initiation of treatment to exclude effects on proliferation. Supernatants and cell lysates from three independent experiments were pooled prior to SDS-PAGE. Primary antibodies for collagen type-1 (diluted 1∶200; sc-28657, Santa Cruz biotechnology, Santa Cruz, CA, USA), fibronectin (1∶10,000; F3648, Sigma Aldrich, Taufkirchen, Germany), alphaSMA (1∶10,000, M0851, DAKO Cytomation, Hamburg, Germany) and gamma-tubulin (1∶5,000; sc-7396, Santa Cruz biotechnology) were applied overnight at 4°C; secondary antibodies (diluted correspondingly 1∶2,000, 1∶5,000, 1∶5,000, 1∶5,000) were added for one hour at room temperature. Densitometry was performed as described previously [38].

### Statistical analysis

Statistical analysis and graph presentation were performed using GraphPad Prism 4 Software (GraphPad, San Diego, CA, USA). For comparisons of the control group versus antagonist groups, one-way analysis of variance (ANOVA) for random measures was applied, followed by Bonferroni's post-hoc test. For comparisons of control PSC versus WIN-treated PSC, a paired t-test was used. ANOVA (and Bonferroni's post-hoc test) was used to compare WIN-treated PSC versus CB-receptor antagonist pre-treated groups. The level of statistical significance was set at p<0.05.

## Results

### Regulation of CB1, CB2 and endocannabinoids in normal pancreas and chronic pancreatitis

**Figure 1:**
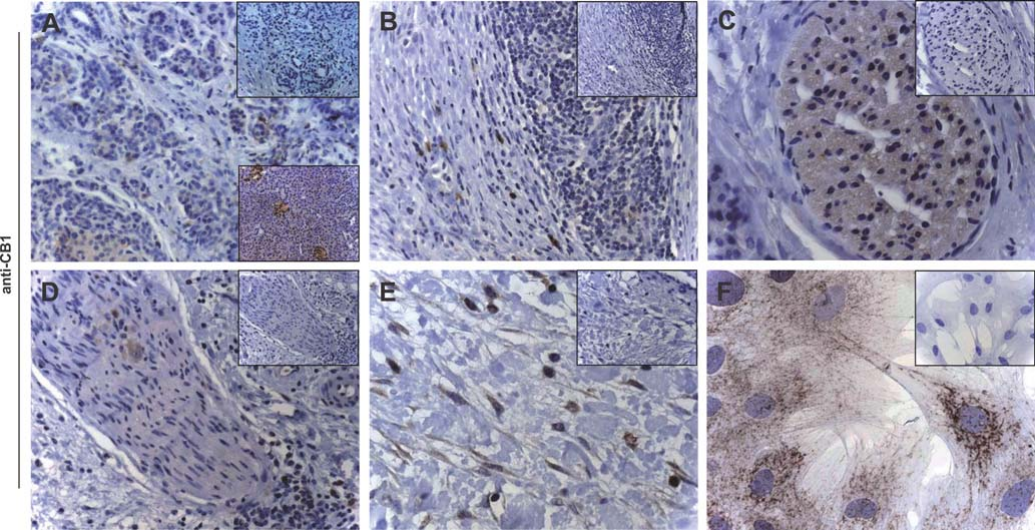
Cannabinoid receptor 1 in human chronic pancreatitis tissues. CB1 immunohistochemistry: staining of tubular complexes (A) but immunonegativity of infiltrating mononuclear cells (B); various staining intensities of intrapancreatic nerves (C&D); pancreatic stellate cells in areas of fibrosis (E) or isolated and cultured in vitro (F). Original magnification: x20 (A, B, D), x40 (C), x80 (F). Insets: negative controls. Lower insets (A): CB1 in normal pancreas.

**Figure 2:**
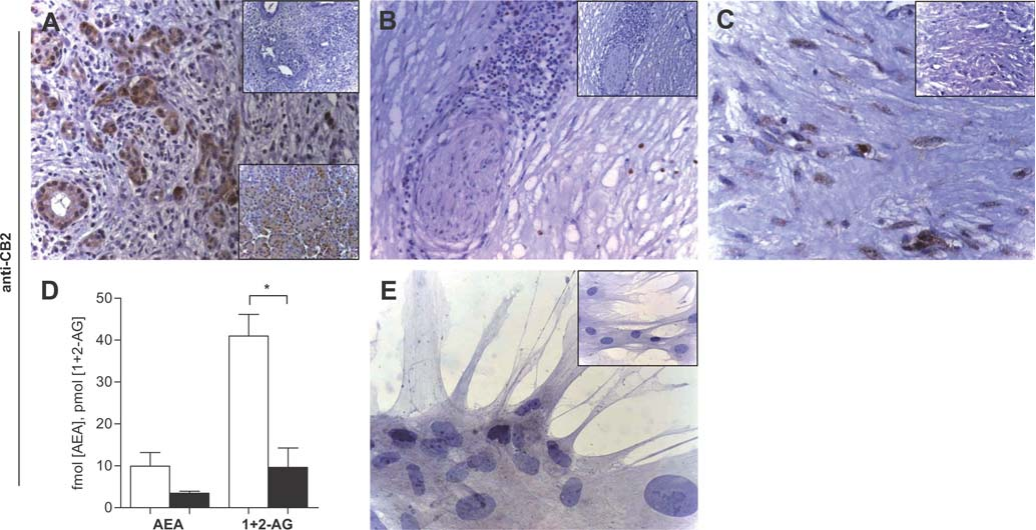
Cannabinoid receptor 2 and endocannabinoid levels in chronic pancreatitis. Using an anti-CB2 antibody, tubular complexes were strongly stained (A). Invading mononuclear cells were also immunopositive (B), whereas intrapancreatic nerves were mostly unstained or only faintly positive (B). Pancreatic stellate cells were immunopositive for CB2 (C). The endocannabinoids anandamide (AEA) and 1+2-arachidonoylglycerol (1+2-AG) were lower in chronic pancreatitis (AEA: p = 0.14 and 1+2-AG: p = 0.0066; D). Cultured pancreatic stellate cells were faintly CB2-immunopositive (E). Original magnification: x20 (A, B), x40 (C), x80 (F). Insets: negative controls. Lower insets (A): CB2 in normal pancreas.

In chronic pancreatitis tissue samples, various staining patterns were present. In tubular complexes which result from metaplasia of acinar cells to duct-like structures, weak immunoreactivity was seen for CB1 (Figure 1A), whereas moderate to strong stainings were observed for CB2 (Figure 2A). Weak immunoreactivity for CB1 in nerves (Figure 1D) was observed in 13 tissue samples, moderate staining in 21 samples, and strong immunostaining in 8 samples (Figure 1C). Immune cell infiltrates were only faintly stained with CB1 (Figure 1B), whereas for CB2, only weak immunoreactivity was seen in nerves (Figure 2B). However, immune cell infiltrates were CB2-positive: in 11 samples there was weak staining (Figure 2B), 18 samples were moderately immunopositive, and 13 samples were strongly stained. Pancreatic stellate cells in areas of fibrosis were also immunopositive for CB1 and CB2 (Figures 1E and 2C). Immunohistochemistry of normal human pancreatic tissues revealed weak immunoreactivity for CB1 and CB2 in pancreatic acini, nerves, blood vessels and ductal cells, as previously described (lower insets Figures 1A & 2A;[39]).

We then assessed the levels of endocannabinoids in chronic pancreatitis. In contrast to the findings in acute pancreatitis, where upregulation of endocannabinoids was observed [39], levels of anandamide were decreased 2.2-fold in chronic pancreatitis (statistically not significant, p = 0.14; Figure 2D) and 1+2-AG levels were reduced 4.2-fold (p = 0.0066; Figure 2D).

### Cannabinoid receptors are expressed on pancreatic stellate cells

Immunocytochemistry revealed that both CB1- and CB2-receptors are present on pancreatic stellate cells (Figures 1F & 2E). While CB1 showed a dotted expression pattern with strong staining intensity (Figure 1F), CB2 immunostaining was less intensely distributed within the stellate cells (Figure 2E).

### Functional significance of the endocannabinoid system in chronic pancreatitis

**Figure 3:**
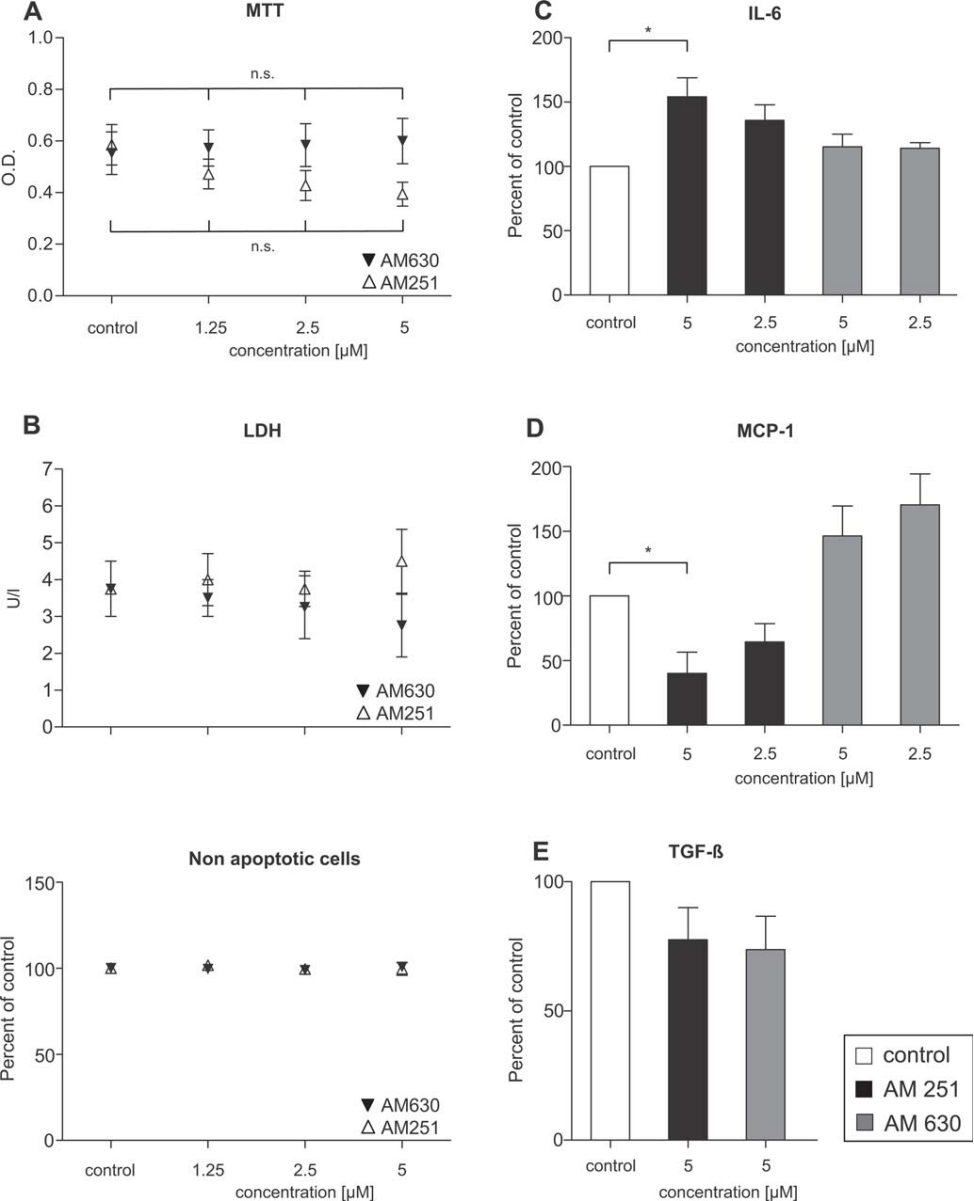
Effects of cannabinoid receptor antagonism on pancreatic stellate cells. (A) MTT assays after 48 hours' incubation of PSC with graded concentrations of CB1- and CB2-receptor antagonists AM251 and AM630 (p = n.s. for concentrations of 1.25, 2.5 and 5 µM). (B) LDH in cell culture supernatants and the fraction of apoptotic cells as judged by the GuavaNexin™ test. AM251 significantly increased IL-6 (C) but decreased MCP-1 secretion (D); AM630 had no effect on IL-6 (C) but induced a tendency towards increased MCP-1 levels (D). CB1-/2-receptor antagonism did not affect TGFbeta (E). Data are shown as mean±SEM (*, p<0.05).

To evaluate the functional relevance of decreased endocannabinoid release and upregulation of cannabinoid receptors in human chronic pancreatitis, CB1 and CB2 receptors were blocked on pancreatic stellate cells (the major contributors of extracellular matrix protein production; all antagonist results from three independently performed experiments) which had been isolated from human chronic pancreatitis tissues. AM251 and AM630 were used as specific antagonists to CB1 and CB2 receptors, respectively. Effects of antagonist treatment on stellate cell proliferation were assessed using MTT tests: both AM630 and AM251 did not influence proliferation of PSC at the tested concentrations of 1.25, 2.5 and 5 µM (p>0.05, respectively, as tested by ANOVA and post-hoc Bonferroni's test; Figure 3A).

Potential effects of cannabinoid receptor antagonism on either necrosis or apoptosis were determined by analyzing levels of lactate dehydrogenase (LDH) in cell culture supernatants and by performing the Guava Nexin™ apoptosis test on AM251- and AM630-treated PSC. These experiments revealed that AM251 and AM630 induced neither necrotic cell death—as judged by unchanged LDH levels—nor apoptosis (Figures 3B). Further analysis of the very low number of apoptotic cells (both in control and antagonist groups) showed that there was no difference in the number of early and late stage apoptotic cells, as well (not shown).

To further assess functional consequences of endocannabinoid blockade in PSC, production of cytokines IL-6 and TGFbeta, chemokine MCP-1 and ECM proteins collagen1 and fibronectin was analyzed. While AM251 significantly induced IL-6 secretion at a concentration of 5 µM (p<0.05, ANOVA; Figure 3C), an inverse effect was observed regarding MCP-1, with AM251 decreasing its levels in cell culture supernatants (p<0.05, ANOVA; Figure 3D). AM630 did not influence IL-6 levels; however, there was a trend toward increased MCP-1 secretion (Figure 3C&D).

**Figure 4:**
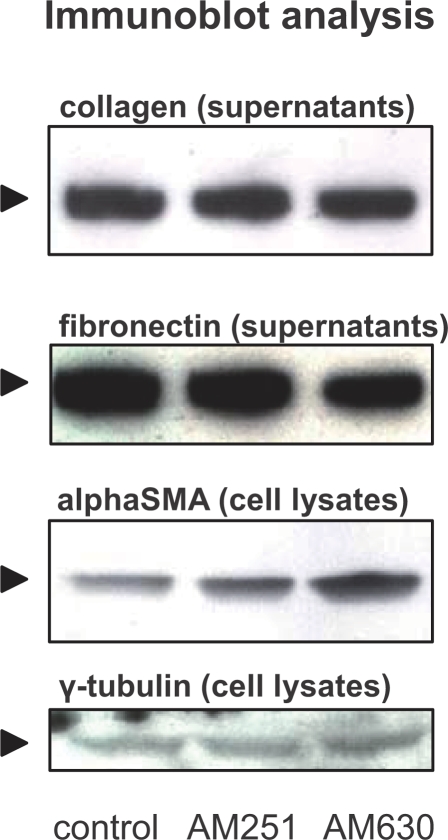
Cannabinoid receptor blockade alters synthesis of ECM proteins. As assessed by immunoblot analysis of cell culture supernatants, AM251 and AM630 (at 2.5 µM) did not affect collagen and fibronection secretion. In PSC cell lysates, alphaSMA levels remain unchanged following CB1-receptor antagonism whereas AM630 induced increased alphaSMA protein levels. White bars: control; black bars: AM251; grey bars: AM630. Data are shown as mean±SEM.

In a next step, the profibrogenic cytokine TGFbeta, which stimulates the synthesis and secretion of fibronectin, collagen1 and matrix metalloproteinases, was analyzed, revealing that neither AM251 nor AM630 significantly altered secreted TGFbeta levels (Figure 3E). Furthermore, AM215 had no effects on fibronectin and collagen 1 secretion (cell culture supernatants of cells grown to 100% confluency before initiation of treatment; immunoblots from three pooled experiments; Figure 4). Blockade of the CB2-receptor by AM630 did also not change collagen 1 and fibronectin production (Figure 4). However, AM630 induced an increase of alphaSMA protein levels (pooled cell lysates from three independently performed experiments; Figure 4).

**Figure 5:**
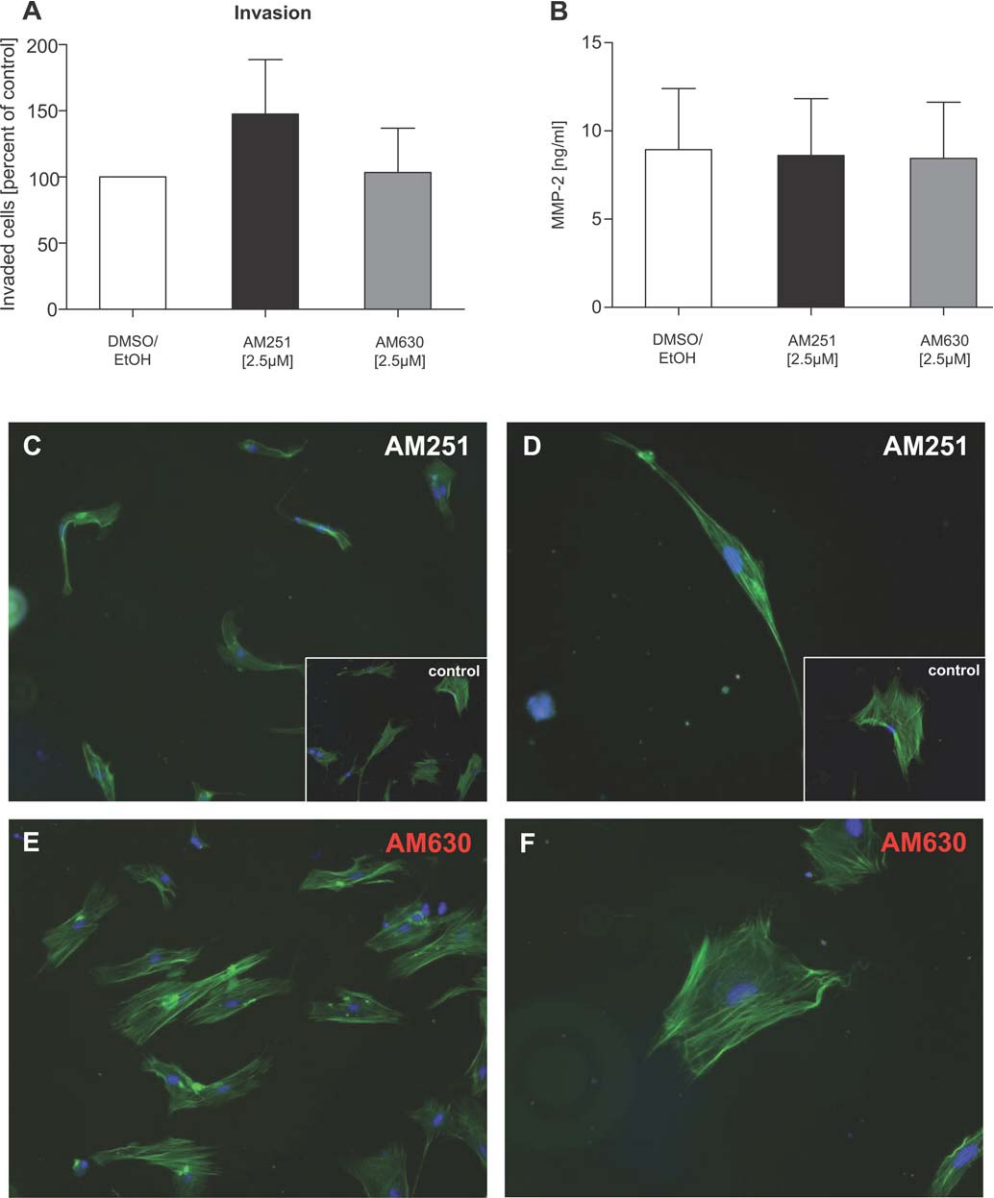
AM251 induces changes in PSC phenotype. (A) Invasion assays demonstrated a trend toward increased invasiveness induced by AM251. (B) MMP-2 levels were unchanged by antagonist treatment. (C&D) AM251-treated pancreatic stellate cells were smaller and thinner, with a more stretched shape, an increased length, and a loss of intracellular fibres (control PSC: insets and Figure 8C&D). Cells treated with AM630 (E&F) closely resembled the control PSC (see insets C&D and Figure 8C&D). Original magnification: x 40 (C&E), x 80 (D&F). Nuclear stain: DAPI. White bars: control; black bars: AM251; grey bars: AM630. Data are shown as mean±SEM.

Evaluation of invasiveness as another key feature of pancreatic stellate cells was carried out using Matrigel™-coated cell culture inserts, as previously described[38]. Although there was a tendency toward an increased number of invaded cells treated with AM251 (p = n.s.; mean increase compared with control: +47%; Figure 5A), MMP-2 levels in cell culture supernatants were not different from the control treatment group (Figure 5B). However, there were distinct changes in stellate cell morphology after incubation with the CB1-receptor antagonist AM251, as judged by actin cytoskeleton staining (Figure 5C&D; control PSC: inserts), whereas AM630-treated PSC closely resembled the control cell phenotype (Figure 5E&F). Treatment of PSC with AM251 induced a thinner, more stretched shape in the cells, with an increased length but an overall smaller size (Figure 5 C&D). Additionally, we observed a marked loss of intracellular fibres upon incubation with AM251. These results are supported by a recent publication which reports on an association between loss of actin polymerization and increased invasiveness [40].

### Cannabinoid receptor activation reduces markers of inflammation and fibrosis in PSC

**Figure 6:**
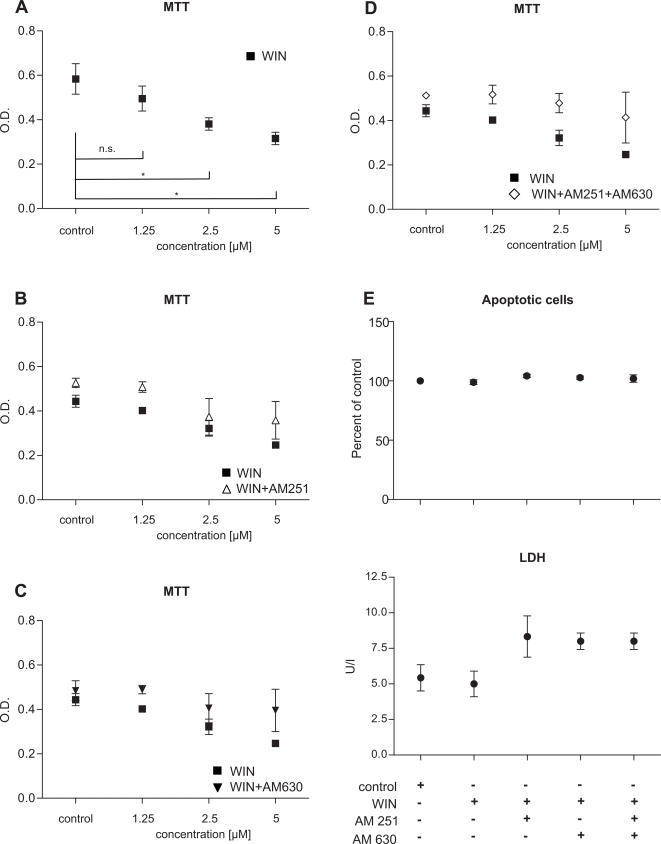
Cannabinoid receptor activation on PSC reduces growth, independent of apoptosis or necrosis. (A) MTT tests of WIN-treated PSC revealed dose-dependent inhibition of growth (p<0.05 at concentrations of 2.5 and 5 µM). (B&C) While AM251 and AM630 alone were not effective, pre-treatment with a combination of AM251 and AM630 blocked WIN-induced growth inhibition (D). The reduction in proliferated cells was neither due to necrosis (unchanged LDH levels) nor to apoptosis (constant low number of apoptotic cells in treated versus control PSC; E). Data are shown as mean±SEM (* p<0.05).

In an approach to reduce the overt activity of stellate cells present in chronic pancreatitis through augmentation of the down-regulated endocannabinoid system, we treated PSC with the synthetic cannabinoid receptor agonist WIN55,212-2 (WIN; all results from three independently performed experiments). Cannabinoid receptor selectivity of the effects of WIN on PSC was evaluated by pre-incubation with the respective antagonists at the cannabinoid-1 and -2 receptors and with a combination of both. As shown by MTT assays, WIN dose-dependently decreased proliferation of PSC (p<0.05 for concentrations of 2.5 µM and 5 µM, respectively, ANOVA), which was partially reversible by pre-incubation with a combination of the CB1- and CB-2-receptor antagonists AM251 and AM630 (Figure 6A–D), but not by the respective receptor antagonists alone (Figure 6B&D). To evaluate whether this effect was due to induction of apoptosis or necrosis, lactate dehydrogenase was analyzed in cell culture supernatants and a GuavaNexin™ test was performed. These experiments revealed that the growth-inhibitory effects of WIN were not due to necrotic cell death, as shown by unchanged LDH levels following WIN treatment with/without pre-incubation with the CB1/2-receptor antagonists (Figure 6E). Similarly, we observed no induction of apoptosis (Figure 6E), either at an early or a late stage (not shown).

**Figure 7:**
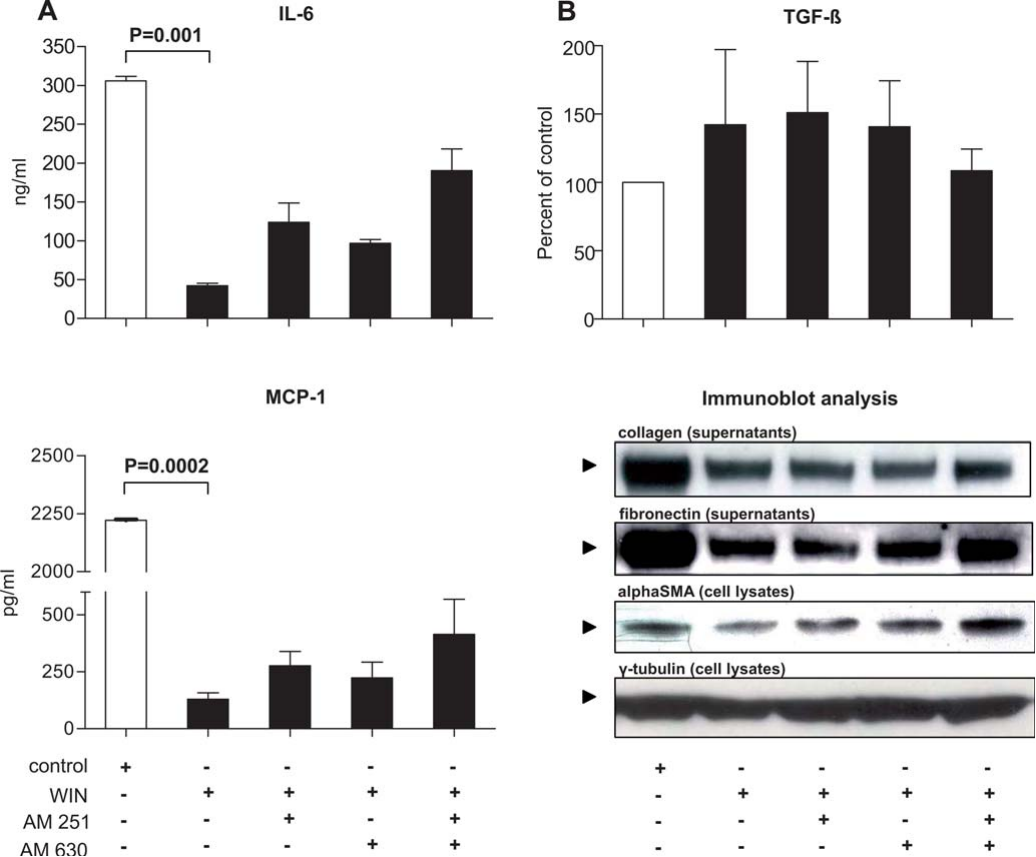
WIN de-activates pancreatic stellate cells. (A) IL-6 and MCP-1 secretion were significantly reduced by WIN (p = 0.001 and p = 0.0002, respectively), independent of TGFbeta (B; unchanged TGFbeta levels). The reduction in IL-6 and MCP-1 levels was partially reversed by a combination of the CB1-receptor and CB2-receptor antagonists AM251 and AM630 (A). While control PSC secreted significant amounts of fibronectin and collagen 1 (as seen by an intense signal at 220 and 190 kDa, respectively), treatment with WIN reduced the signal at the respective molecular weights (B; immunoblots of cell culture supernatants, pooled from three independent experiments). AlphaSMA levels were also suppressed by WIN (B; immunoblot of PSC cell lysates; gamma-tubulin: equal loading control). A combination of both antagonists AM251 and AM630 partially reversed the suppressive effects elicited by incubation with WIN (B, immunoblots of fibronectin, collagen 1 and alphaSMA). White bars: control; black bars: WIN55,212-2±AM251/AM630. Data are shown as mean±SEM.

To assess the effects of WIN treatment of PSC on the secretion of cytokines IL-6, MCP-1 and TGFbeta, cell culture supernatants were subjected to ELISA. These experiments demonstrated that IL-6 and MCP-1 secretion were significantly reduced by WIN (p = 0.001 and p = 0.0002, respectively; Figure 7A). Preincubation with a combination of AM251 and AM630 (5 µM, respectively) partially reversed this effect (Figure 7A). In contrast, there was a trend toward increased TGFbeta levels (increase by 42%; Figure 7B) which was nearly absent when cells had been pre-treated with a combination of AM251 and AM630 (increase by only 8%; Figure 7B). Immunoblot analysis of cell culture supernatants showed significantly reduced fibronectin and collagen 1 levels after WIN treatment (supernatants of PSC grown to 100% confluency before initiation of treatment; Figure 7B). Pre-incubation with AM251 and/or AM630 (5 µM) revealed that the suppressive effect of WIN on fibronectin and collagen 1 production was partially reversible only when using both antagonists concomitantly (Figure 7B; pooled cell culture supernatanty from three independently performed experiments). Furthermore, WIN induced a reduction in alphaSMA levels which was also partially reversed by pre-incubation with a combination of both CB-receptor antagonists (Figure 7B; equal loading: gamma-tubulin).

### Cannabinoids decrease invasiveness of PSC and downregulate MMP-2

**Figure 8:**
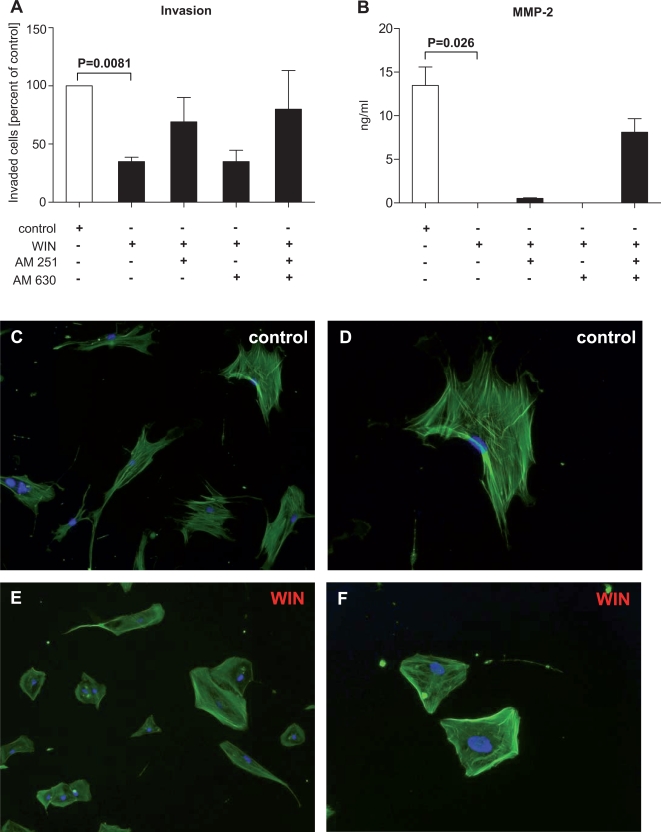
Cannabinoid receptor activation reduces invasiveness. A significant reduction in the number of invaded cells was seen after treatment with WIN (p = 0.0081; A). A near-complete reversal of the reduced invasiveness was seen when the cells were pre-incubated with AM251 or a combination of AM251 and AM630 (A). This was not found with AM630 alone (A). WIN induced a significant decrease in MMP-2 levels (p = 0.026; B) which was partially reversible by a combination of AM251 and AM630 (B). Actin cytoskeleton staining with phalloidin demonstrated that control PSC showed the normal cellular structure (C&D) but that WIN induced a more round and smaller PSC phenotype (E&F). Original magnification: x 40 (C&E), x 80 (D&F). Nuclear stain: DAPI. White bars: control; black bars: WIN55,212-2±AM251/AM630. Data are shown as mean±SEM.

As with CB receptor antagonists alone, invasiveness of PSC following treatment with WIN (+/− pre-incubation with AM251 and/or AM630) was analyzed using Matrigel™ invasion chambers (results from four independent experiments which were performed in duplicates). WIN significantly reduced the number of invaded cells (p = 0.0081; Figure 8A), whereas pre-incubation with AM251 or a combination of AM251 and AM630 partially reversed this effect (5 µM, respectively; Figure 8A). Pre-treatment with AM630 showed no differences from treatment with WIN alone (Figure 8A).

To evaluate whether the WIN-induced reduction in invasiveness was due to decreased levels of matrix metalloproteinase 2, cell culture supernatants were analyzed by ELISA after WIN treatment (+/− pre-incubation with AM251/AM630, 10 µM). We observed a significant decrease of MMP-2 levels after treatment with WIN (p = 0.026; Figure 8B) which was specifically reversible using a combination of both antagonists (to >50% of control cell culture supernatants).

Analysis of the PSC morphology after treatment with WIN (as shown by actin cytoskeleton immunofluorescence) demonstrated distinct changes: whereas cell morphology was unchanged in control PSC (Figure 8C&D), WIN induced a more round-shaped phenotype, with a reduction in cellular extensions (Figure 8E&F). We also observed a loss of intracellular fibres, accompanied by a disorganization of these fibres and a generally smaller PSC phenotype. Furthermore, the number of cell-cell contacts tended to be lower after treatment with WIN (Figure 8E).

## Discussion

In the present study, we show that activation of the endocannabinoid system in chronic pancreatitis-derived stellate cells specifically induced a more quiescent phenotype, accompanied by suppression of pro-inflammatory cytokines and extracellular matrix proteins as well as a decrease in invasiveness of PSC. The loss of functional pancreatic parenchyma, which is substituted by a fibrotic scar with massive infiltration of lymphocytes, is characteristic of chronic pancreatitis pathobiology. So far, there is no treatment available for controlling the excessive activation of pancreatic stellate cells, which mainly produce the extracellular matrix and which activate themselves in an autocrine loop by secreting pro-fibrogenic molecules such as TGFbeta and by producing pro-inflammatory cytokines.

The most important finding of this study is that cannabinoid receptor activation induces a quiescent phenotype of chronic pancreatitis-derived PSC by downregulating production of extracellular matrix proteins and inflammatory cytokines. This effect was accompanied by marked changes in PSC appearance toward a less mesenchymal-like phenotype. Since migration of PSC to sites of damage can induce either re-differentiation of PSC and tissue repair or support activation and fibrosis, we evaluated the invasive potential as a factor contributing to PSC motility[12], [13], [41], [42]. We observed that suppression of PSC invasiveness by unselective CB receptor activation was accompanied by decreased MMP-2 levels. This could be particularly important not only in terms of invasiveness but also for influencing the distorted balance of matrix synthesis and degradation, since it is known that increased levels of MMP-2 (not only in chronic pancreatitis) facilitate the deposition of pathological fibrillar collagen[12], [43]. Furthermore, it has been suggested for both pancreatic and hepatic stellate cells that MMP-2 may have effects on proliferation. Thus, cannabinoid-induced suppression of MMP-2 could be a mediator of the induction of a more quiescent phenotype, leading to reduced collagen synthesis.

Furthermore, there are a number of regulators of PSC activation: 1) paracrine factors such as cytokines (IL-1, MCP-1), growth factors (TGFbeta and PDGF) and endothelium-derived substances (endothelin-1) which are released by recruited inflammatory cells; and 2) factors secreted by destroyed parenchymal cells, such as acinar, endothelial and ductal cells [14], [15], [44]–[46]. PSC can also perpetuate their activation by producing autocrine mediators such as IL-6, TGFbeta, PDGF or the most recently discovered periostin [38]. Once activated, PSC recruit and stimulate leukocytes via MCP-1 and IL-8. In our experimental setup, we chose to analyze MCP-1 levels upon blockade or augmentation of the endocannabinoid system with synthetic cannabinoids. Here, significantly reduced levels of MCP-1 and IL-6 were observed, suggesting interference of cannabinoids in the autocrine loop, with the effect of de-sensitization of excessively stimulated pancreatic stellate cells. These results are supported by the observation that the endocannabinoid system is suppressed in chronic pancreatitis, which is particularly important since chronic pancreatitis—in contrast to liver cirrhosis—is usually associated with a severe pain syndrome. Thus, down-regulation of pain-inhibitory endocannabinoids could participate in pain generation in chronic pancreatitis. In concordance with the contradictory results of cannabinoid receptor activation and antagonism in hepatic stellate cells, blockade of the remaining ECS activity exerted at least some beneficial effects, such as suppression of the chemokine MCP-1 which may be explained by increased endocannabinoid secretion upon blocking the receptors. However, we also observed that cannabinoid receptor-1 antagonism induced production of the pro-inflammatory cytokine IL-6 and induced a more motile PSC phenotype, as judged by a trend toward increased invasiveness and a longitudinally stretched appearance. For liver cirrhosis it has recently been shown that CB2-receptor activation mediated anti-fibrosis by apoptosis induction and growth inhibition of hepatic stellate cells [33]. In our study, antagonism of cannabinoid receptor-2 elicited no such effects.

Because drugs for the treatment of chronic pancreatitis should ideally exert anti-fibrotic and anti-inflammatory properties, their bimodal effects rather contradict a therapeutic use of CB-receptor antagonists and promote the hypothesis that (re-)activation of the (endo-)cannabinoid system in chronic pancreatitis may be beneficial for suppressing disease progress. This holds particularly true since the suppressive effects induced by the cannabinoid receptor agonist WIN55212,2 were reversible by pre-incubation with a combination of CB1- and CB2 receptor antagonists AM251 and AM630. Though CB1 receptors constitute the “main primary antinociceptive targets for systemically- or peripherally-applied cannabinoids in vivo” [47], activation of central CB1 receptors also dose-dependently induces side-effects such as hindrance of activity, motor dysfunction, sedation or catalepsy [47]–[49]. Thus, a major challenge for the clinical use of cannabinoids, i.e. as an anti-fibrogenic therapy, will be the reduction of these cognitive, affective and motor function side-effects [47], [50]. An alternative to selective, peripheral cannabinoid receptor agonists might be the manipulation of endocannabinoid metabolism by blockade of endocannabinoid degradation or uptake [50], [51]. This approach could be particularly advantageous in chronic pancreatitis where the endocannabinoid system is locally suppressed and where this pathological state could thus be reversed. However, future studies are needed to pinpoint the precise mechanisms of cannabinoid-induced de-activation of pancreatic stellate cells and particularly its effects in vivo: 1) in a disease-prevention model, 2) in an approach to slow down the destruction of functional pancreatic parenchyma, and 3) in their potency to reverse fibrosis.

In conclusion, we show that the endocannabinoid system is downregulated in chronic pancreatitis and that its augmentation via exogenously administered cannabinoids specifically reduces activation of pancreatic stellate cells. These experiments lay a basis for testing the value of synthetic cannabinoids in the treatment of chronic pancreatitis.
